# Comprehensive immunological profiling of acute ischemic stroke during mechanical thrombectomy: myeloid cell activation and molecular signatures in blood and thrombus

**DOI:** 10.3389/fstro.2026.1731953

**Published:** 2026-02-12

**Authors:** Wirginia Krzyściak, Tadeusz Popiela

**Affiliations:** 1 Department of Medical Diagnostics, Faculty of Pharmacy, Jagiellonian University Medical College, Kraków, Poland; 2 Chair of Radiology, Jagiellonian University Medical College, Kraków, Poland; 3 Department of Diagnostic Imaging, University Hospital, Kraków, Poland

**Keywords:** acute ischemic stroke, blood biomarkers, immunology, neutrophils, thrombectomy, thrombus, transcriptomics, recanalization success

## Abstract

**Background:**

Acute ischemic stroke (AIS) induces a complex local and systemic inflammatory response; however, most studies rely solely on peripheral blood, providing an incomplete view of immune activity at the occlusion site and within the thrombus.

**Objective:**

To characterize immune activation and transcriptomic signatures of myeloid cells across three compartments—arterial blood at the occlusion site, peripheral blood, and thrombus—and to evaluate their associations with radiological and clinical outcomes following mechanical thrombectomy.

**Methods:**

This prospective, single-center study will include AIS patients treated with mechanical thrombectomy. Matched arterial, peripheral, and thrombus samples will undergo spectral flow cytometry, cytokine profiling, cell-free DNA (cfDNA) quantification, microscopy, and RNA sequencing. Immune and molecular readouts will be correlated with clinical scores (NIHSS, mRS), imaging markers (e.g., hyperdense middle cerebral artery sign [HMCAS]), and procedural outcomes (TICI score, number of passes).

**Significance:**

Integrating local and systemic immune profiles with clinical and radiological parameters may identify biomarkers predictive of thrombectomy efficacy and functional recovery, thereby supporting precision-medicine approaches in AIS.

**Clinical trial registration:**

www.ClinicalTrials.gov

## 1 Introduction

Acute ischemic stroke (AIS) remains a major cause of disability despite advances in reperfusion therapies (GBD 2021 Stroke Risk Factor Collaborators, 2021). Mechanical thrombectomy improves recanalization (Oliveira et al., 2022; Liu et al., 2020); however, patient outcomes vary widely, suggesting a role for secondary inflammatory mechanisms (Shen et al., 2023; Lattanzi et al., 2021; Aly et al., 2021). Myeloid cells, including neutrophils and monocytes, contribute to tissue injury, thrombosis, and reperfusion success, yet most studies focus exclusively on peripheral blood, overlooking immune processes occurring directly at the occlusion site and within the thrombus (Lin et al., 2025; Cao et al., 2024; Tsalta-Mladenov and Andonova, 2024; Otsu et al., 2023).

This gap limits understanding of local immunopathology and its relevance to clinical outcomes (Li et al., 2023; Liu et al., 2023; Li et al., 2025). The aim of this study is to compare immune profiles and transcriptomic signatures of myeloid cells across three compartments—arterial blood, peripheral blood, and thrombus—and to examine their associations with radiological markers and clinical recovery following thrombectomy.

This approach addresses a critical missing link between localized immune activation and patient prognosis, providing a framework for identifying biomarkers relevant to personalized stroke care.

## 2 Methods and design

### 2.1 Study design and objectives

This is a single-center, observational, translational study with both prospective and retrospective components, designed to investigate local and systemic immune mechanisms in acute ischemic stroke (AIS) patients treated with mechanical thrombectomy (MT).

The primary objective is to characterize the immunophenotypic and transcriptomic signatures of myeloid cells across three biological compartments: (i) arterial blood aspirated at the site of vessel occlusion, (ii) peripheral venous blood, and (iii) retrieved thrombus material.

Secondary objectives include:

1. Correlating immune and molecular markers with radiological features, including Hounsfield unit (HU) values and the presence of the hyperdense middle cerebral artery sign (HMCAS);
2. Assessing associations with procedural and clinical outcomes, including the Thrombolysis in Cerebral Infarction (TICI) score and the 90-day modified Rankin Scale (mRS);
3. Comparing local (arterial and thrombus-derived) vs. systemic (peripheral) immune activation to identify biomarkers predictive of reperfusion success and functional recovery.

### 2.2 Study setting and timeline

The study will be conducted at the Emergency Radiology Unit, the Angiography and Interventional Radiology Unit, and the Department of Neurology at the University Hospital in Krakow. All study protocols have been approved by the Jagiellonian University Bioethics Committee.

The retrospective arm includes archived thrombus samples collected during the 2024–2025 pilot phase.

The prospective arm includes patient enrollment and biospecimen collection from July 2025 to December 2027, with follow-up completed by December 2029.

A schematic workflow distinguishing the retrospective and prospective components is provided in Table 1.

**Table 1:** Study workflow and sample collection strategy.

**Study phase**	**Study type**	**Patient population**	**Biological material**	**Time point**	**Main analyses**
Pilot phase (2024–2025)	Retrospective	AIS patients previously treated with MT	Archived thrombus	Post-procedure (archived)	Histology, IHC, clot composition
Enrollment	Prospective	AIS with anterior LVO undergoing MT (no IVT)	—	Admission	Eligibility assessment, consent
MT procedure	Prospective	Same cohort	Arterial blood (occlusion site)	During MT	Flow cytometry, RNA-seq, cytokines
MT procedure	Prospective	Same cohort	Retrieved thrombus	During MT	Histology, IHC, NET analysis
MT procedure	Prospective	Same cohort	Peripheral venous blood	During MT	Systemic immune profiling
Follow-up	Prospective	Same cohort	Peripheral venous blood	90 days	Longitudinal immune analyses
Outcome assessment	Prospective	Same cohort	Clinical and imaging data	90 days	NIHSS, mRS, TICI, imaging correlations
Data integration	Combined	Full cohort	Multimodal dataset	Final analysis	Integrated clinical–radiological–immunological modeling

Molecular analyses of blood samples will be performed at the Department of Medical Diagnostics and the Department of Cytobiology of the Chair of Pharmacobiology at the Faculty of Pharmacy, Jagiellonian University Medical College.

### 2.3 Study population

Eligible participants are adult patients (≥18 years) with acute ischemic stroke (AIS) due to anterior circulation large-vessel occlusion undergoing mechanical thrombectomy (MT).

To ensure cohort homogeneity and minimize confounding in molecular analyses, patients receiving intravenous thrombolysis (IVT) prior to MT are excluded.

### 2.4 Rationale for exclusion of intravenous thrombolysis

Patients treated with intravenous thrombolysis (IVT) are excluded because thrombolytic agents profoundly alter thrombus structure, systemic cytokine levels, neutrophil activation, and neutrophil extracellular trap (NET) formation. These effects may confound the interpretation of local and systemic immune signatures, as well as their association with radiological features and intrinsic clot biology.

### 2.5 Imaging assessment and inter-rater reliability

Non-contrast computed tomography (CT) is performed immediately prior to mechanical thrombectomy (MT). Hounsfield unit (HU) measurements are obtained using standardized regions of interest (ROIs).

HU measurements and assessment of the hyperdense middle cerebral artery sign (HMCAS) are performed independently by two board-certified neuroradiologists blinded to clinical and histological data.

Interobserver discrepancies greater than 10 HU are resolved by consensus.

### 2.6 Outcome assessment and bias control

Angiographic reperfusion is graded using the Thrombolysis in Cerebral Infarction (TICI) scale by two independent interventional neuroradiologists blinded to immunological data.

Functional outcome, assessed using the modified Rankin Scale (mRS) at 90 days, is evaluated by certified neurologists not involved in the mechanical thrombectomy (MT) procedure, either during outpatient visits or through standardized telephone interviews.

#### 2.6.1 Inclusion criteria

Eligible participants are adults (≥18 years) with acute ischemic stroke caused by an anterior circulation large-vessel occlusion, confirmed by non-contrast computed tomography (CT) and digital subtraction angiography, who undergo mechanical thrombectomy with successful thrombus retrieval and arterial blood sampling at the occlusion site. Peripheral venous blood sampling and clinical follow-up, including assessment of the 90-day modified Rankin Scale (mRS), must be feasible. Written informed consent is obtained from the patient or a legal representative; documented oral consent is acceptable when written consent cannot be obtained.

This criterion reflects technical feasibility and is addressed as a potential source of selection bias in the Study Limitations section.

#### 2.6.2 Exclusion criteria

Patients are excluded if they: (i) received intravenous thrombolysis prior to mechanical thrombectomy; (ii) have contraindications to mechanical thrombectomy according to current clinical guidelines; (iii) lack technical feasibility for arterial blood sampling at the occlusion site; or (iv) have inadequate imaging quality precluding Hounsfield unit (HU) analysis.

Patients treated with intravenous thrombolysis were excluded as detailed in Section 2.4.

Imaging assessment was performed as described in Section 2.5.

### 2.7 Biospecimen collection, processing, and analytical workflow

Biological material will be collected prospectively during mechanical thrombectomy in patients with acute ischemic stroke due to large vessel occlusion, following written informed consent. Biospecimen acquisition is fully integrated into standard endovascular procedures and does not alter clinical management.

#### 2.7.1 Types, sequence, and sources of biospecimens

Biological material will be obtained in parallel from three complementary sources: arterial blood, peripheral venous blood, and embolic material retrieved during mechanical thrombectomy. Arterial blood is aspirated directly at the site of occlusion, whereas peripheral venous blood is collected from the cubital vein. Embolic material (thrombus) is collected immediately after retrieval.

The sequence, type, volume, and downstream use of all collected biospecimens are summarized in Table 2.

**Table 2:** Sequence, type, volume, and downstream use of collected biospecimens.

**Material source**	**EDTA tubes**	**Serum tubes**	**Formalin (4% buffered)**	**Downstream use**
Arterial blood proximal to occlusion	2 mL	2 mL	–	Flow cytometry, hematology
Arterial blood distal to occlusion	2 mL	2 mL	–	Flow cytometry, hematology
Peripheral venous blood (cubital vein)	2 mL	2 mL	–	Flow cytometry, serum cytokine analysis
Embolic material (thrombus)	–	–	0.5–1.0 cm	50% histopathology; 50% isolation and immunophenotyping of myeloid cells (including neutrophils)

#### 2.7.2 Arterial and peripheral blood sampling

Arterial blood samples are collected immediately prior to clot retrieval using the same aspiration catheters and microcatheters employed during the thrombectomy procedure. Sampling is performed under fluoroscopic guidance after confirmation of vessel occlusion by CT, CTA, CTP, and digital subtraction angiography (DSA).

Proximal and distal arterial samples are obtained using aspiration catheters (5F or 6F) and microcatheters with an inner diameter of approximately 0.53 mm and a length of 150 cm. To minimize dilution and contamination, the catheter dead space is discarded before sample collection. Peripheral venous blood is drawn simultaneously from the cubital vein.

For each patient, a total of six blood collection tubes are obtained: three EDTA tubes and three serum tubes, each with a volume of 2 mL from each sampling site.

Selected cytokines are measured in serum samples. Blood collected into serum tubes is centrifuged according to standardized protocols, and the resulting serum is aliquoted and stored at −80 °C until further analysis using validated analytical platforms.

Only patients with successful recanalization (TICI ≥ 2b) are included in arterial sampling to ensure procedural safety and sample integrity. This inclusion criterion is acknowledged as a potential source of selection bias and will be addressed in the statistical analysis.

#### 2.7.3 Thrombus handling and downstream analyses

Retrieved embolic material is processed immediately after extraction. Each thrombus specimen is divided into two equal parts. One half is fixed in 4% buffered formalin for histopathological analysis, including assessment of thrombus architecture and spatial immune cell distribution. The second half is transferred into a viability-preserving transport medium for isolation of thrombus-infiltrating cells.

Cell isolation is performed using a standardized mechanical–enzymatic dissociation protocol with collagenase I/IV and DNase I under continuous microscopic control. Cell viability is assessed using Trypan Blue exclusion and Annexin V/PI staining. The entire workflow—from thrombus retrieval to initiation of downstream cellular analyses—does not exceed 3 h, minimizing *ex vivo* cell activation.

In parallel, real-time hematological analysis is performed using an automated analyzer, complemented by manual leukocyte differential assessment.

### 2.8 Clot histopathology and immunophenotyping

Retrieved thrombi are fixed, paraffin-embedded, and analyzed using hematoxylin and eosin (H&E) and Martius Scarlet Blue (MSB) staining to assess overall structure and major components, including red blood cells (RBCs), fibrin, and platelets. Immunohistochemistry is performed to identify leukocyte subsets (CD66b, CD3, CD68) and neutrophil extracellular traps (NETs; H3Cit). Quantitative image analysis is conducted using digital pathology software, with results expressed as the proportion of positively stained area relative to the total clot area. NET burden and spatial distribution within thrombi are systematically evaluated.

Preliminary histopathological observations of thrombi analyzed by the project team confirm the presence of extensive, multilayered NET structures stabilizing the thrombus mass, which may account for the stiffness and resistance of mixed thrombi to mechanical extraction.

Thrombi from 30 patients were analyzed using histopathological methods, including H&E and special stains. The results were consistent across methods and indicated that the thrombi were fibrin-rich (“white”), with a dense, multilayered network of fibrin and collagen; partially organized, containing numerous intrathrombus spaces; and containing irregular, scattered neutrophil foci, indicative of their active involvement. They were poor in erythrocytes, distinguishing them from cardiogenic thrombi, and devoid of atherosclerotic material, although they exhibited features of chronic vessel wall inflammation.

These observations confirm the heterogeneity of the thrombus microenvironment and suggest an active and diverse role for neutrophils, supporting further analyses of their phenotype and metabolism.

### 2.9 Immunological objectives

The study aims to characterize myeloid cell activation states, neutrophil extracellular trap (NET) formation, and transcriptomic signatures across arterial blood, peripheral blood, and thrombus compartments. By integrating histology, immunophenotyping, RNA sequencing, and imaging data, the study seeks to identify immune biomarkers associated with thrombus composition, reperfusion success, and functional recovery following thrombectomy.

### 2.10 Participant recruitment

Patients are recruited at the University Hospital in Krakow by neurologists and interventional radiologists involved in the qualification and performance of mechanical thrombectomy. Eligibility is confirmed immediately prior to the procedure. Recruitment procedures follow Good Clinical Practice standards, with enrollment monitored monthly by the principal investigators.

## 3 Sample collection and processing

This study will analyze three biological compartments:

1. Arterial blood, collected directly from the site of vessel occlusion using microcatheters or aspiration catheters (1.7F−6F) during the mechanical thrombectomy (MT) procedure;
2. Peripheral venous blood, obtained under controlled clinical conditions;
3. Thrombotic material, retrieved using stent retrievers or aspiration devices during MT.

Peripheral venous blood will be collected at two time points:

- During the MT procedure: Sampling will be performed by an anesthesiologist from the radial vein using vacuum-assisted collection systems. The procedure will be conducted under continuous monitoring of vital signs to ensure patient safety and hemodynamic stability throughout venipuncture and the entire collection process.
- At the 90-day follow-up visit in the neurology outpatient clinic: Peripheral blood will again be collected from the antecubital fossa to allow for longitudinal analyses of systemic biomarkers and immune profiles post-treatment.

All samples will be immediately stabilized using appropriate preservation agents (e.g., RNAlater for nucleic acids, anticoagulants for cellular assays) and transported on ice to the laboratory. Sample processing will begin within 30 min of collection and include:

- Immunophenotyping (e.g., flow cytometry) to characterize leukocyte subsets and activation status,
- RNA extraction for transcriptomic analyses,
- Enzyme-linked immunosorbent assays (ELISA) for quantification of circulating biomarkers and inflammatory mediators, and
- Microscopy, including conventional and confocal imaging for structural evaluation.

These procedures are designed to preserve the cellular integrity and molecular fidelity of all samples, ensuring reproducibility and reliability of downstream analyses. In addition, cytokine profiling will be conducted using the Luminex platform, and transcriptomic findings will be validated through quantitative real-time PCR (qPCR) and TaqMan Low-Density Arrays (TLDA) for broader gene expression analysis.

All clinical, radiological, and molecular data will be integrated to identify correlations between the patients' immunological profiles and treatment outcomes, as measured by the National Institutes of Health Stroke Scale (NIHSS), the modified Rankin Scale (mRS), and the Thrombolysis in Cerebral Infarction (TICI) score.

The study will be conducted between January 2026 and December 2029, based on the methodological framework developed during the 2024–2025 preparatory and pilot phase.

**Figure 1:**
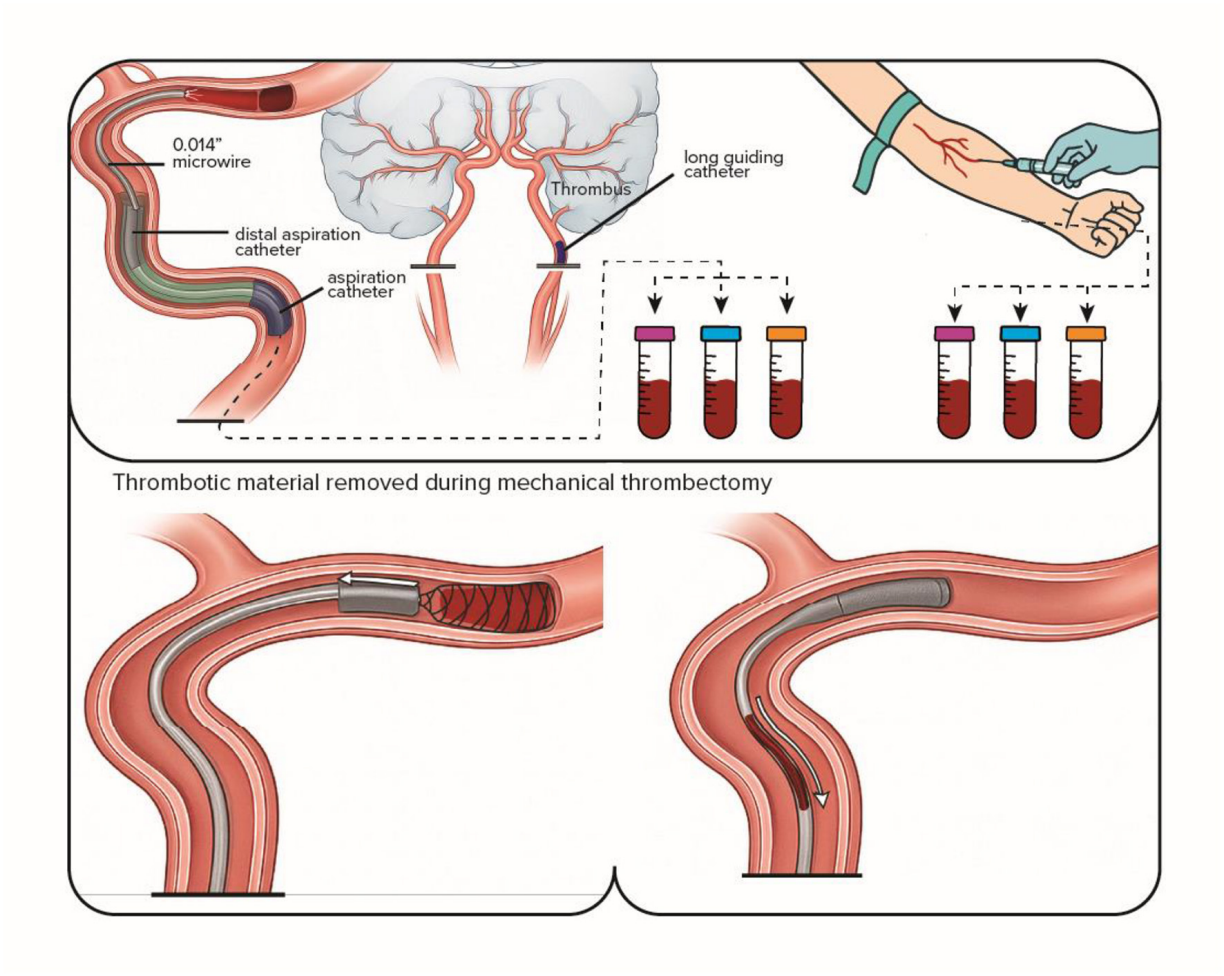
Schematic overview of biospecimen collection during mechanical thrombectomy in patients with acute ischemic stroke. The figure illustrates site-specific sampling of arterial blood obtained at the occlusion site using a dedicated microcatheter, peripheral venous blood collected from the antecubital vein during the procedure, and thrombotic material retrieved from the occluded vessel. Following collection, blood samples are processed in appropriate tubes for downstream hematological and immunological analyses, while embolic material is allocated for immunophenotypic characterization and histopathological evaluation. The workflow further depicts subsequent sample handling, stabilization, and analytical steps leading to integrated immunophenotypic and molecular profiling.

Figure 1 provides a schematic overview of the sample collection and processing workflow, detailing the timing, anatomical source, stabilization, and analytical steps for each biological compartment.

The study workflow, including sample collection, processing, and data integration, is summarized in Figure 1.

## 4 Summary

This multi-phase, multidisciplinary study leverages advanced immunological, molecular, and imaging technologies to comprehensively characterize the immune landscape of AIS in the setting of MT. By integrating clinical, radiological, histological, and multi-omics data—including a retrospective arm focused on archived thrombus tissue—the study aims to uncover robust biomarkers of stroke pathophysiology, therapeutic response, and clinical prognosis. The insights gained may guide the development of personalized treatment strategies and enhance outcomes in acute ischemic stroke care.

### 4.1 Statistical analysis and sample size considerations

#### 4.1.1 Overview and analytical strategy

Statistical analyses were prespecified to align with the primary objective of assessing agreement between leukogram parameters obtained from arterial and peripheral blood samples, as well as secondary objectives exploring associations between hematological markers and clinical and radiological outcomes in patients with acute ischemic stroke undergoing endovascular treatment.

All analyses were two-sided with a predefined significance level of α = 0.05. Given the exploratory and hypothesis-generating nature of the study—particularly for secondary analyses—no formal adjustment for multiple comparisons was applied, in accordance with established methodological recommendations for exploratory biomedical research.

#### 4.1.2 Descriptive statistics

Continuous variables were summarized as mean [standard deviation (SD)], with 95% confidence intervals (CI) calculated using the *t* distribution. Categorical variables were reported as counts (percentages), with 95% CI estimated using the Wilson score method. Variations in sample size across variables reflect missing data and are explicitly reported for transparency.

#### 4.1.3 Agreement between arterial and peripheral blood measurements

The primary analysis of agreement between paired arterial and peripheral leukogram parameters was conducted using Bland–Altman analysis, with bias and limits of agreement calculated according to standard methodology.

Agreement analyses were performed for each leukogram parameter using complete paired observations. Results were visualized using Bland–Altman plots, with additional stratification by sex to explore potential effect modification.

As a complementary method robust to non-normality and outliers, Passing–Bablok regression was applied to evaluate proportional and constant bias between measurement sites. Slopes and intercepts were estimated with 95% CI; proportional bias was inferred if the CI of the slope excluded 1, and constant bias if the CI of the intercept excluded 0.

#### 4.1.4 Correlation analyses

Associations between selected arterial hematological parameters (including NLR, ΔNLR, band granulocytes, reactive lymphocytes, and monocytes) and clinical or imaging outcomes were explored using Spearman's rank-order correlation coefficient (ρ). This nonparametric method was selected due to small sample sizes, potential non-normality, and the possibility of monotonic but non-linear relationships. Correlation strength was interpreted using established thresholds for medical research.

#### 4.1.5 Multivariable modeling

To estimate adjusted associations between hematological parameters and key clinical outcomes while minimizing bias in a small-sample context, doubly robust generalized estimating equation (GEE) models were employed. Each model included a single continuous exposure and adjusted for predefined confounders (age and sex). Robust standard errors were used to account for potential heteroscedasticity and model misspecification. Binary outcomes (mTICI 3 vs. 2b/2c) were modeled using a log link to estimate risk ratios, while continuous outcomes were modeled using an identity link to estimate adjusted mean differences.

#### 4.1.6 Sample size and statistical power

The study analyzed data from 37 patients, reflecting a prospectively collected, single-center cohort and representing a pilot-scale investigation. This sample size was sufficient to estimate agreement parameters (bias and limits of agreement) with acceptable precision for descriptive and exploratory purposes but was not powered to detect small effect sizes in secondary correlation or multivariable analyses.

An a priori power calculation was performed to inform future confirmatory studies. Assuming detection of a moderate Spearman correlation (ρ = 0.30), with a two-sided α = 0.05 and 80% power, the minimum required sample size was estimated at 85 participants using Fisher's *z* transformation. Applying a conservative 15% inflation factor to account for missing data and deviations from distributional assumptions yields a recommended target sample size of approximately 98 participants.

#### 4.1.7 Statistical software

All analyses were conducted using R Statistical Software (version 4.5.2) on Windows 11 Pro (64-bit), utilizing validated packages for agreement analysis, regression modeling, visualization, and reporting, including blandr, mcr, drgee, pwr, ggplot2, and gtsummary (Bland and Altman, 1986; Passing and Bablok, 1983; Schober et al., 2018; Rothman, 1990; Champely, 2020; Bang and Robins, 2005; Lambertsen et al., 2012; Schnabel et al., 2013; Zeger et al., 1988; Chan et al., 2023; Datta, 2024; Jakob, 2023; Makowski et al., 2023; Potapov et al., 2024; R Core Team, 2025; Sjoberg et al., 2021; Wickham, 2007, 2016; Wickham et al., 2023; Zetterqvist and Sjlander, 2015; Zetterqvist et al., 2016).

## 5 Preliminary results

The preliminary results provide a solid methodological and biological foundation for the implementation of the project.

### 5.1 Characteristics of thrombi retrieved during thrombectomy

**Figure 2:**
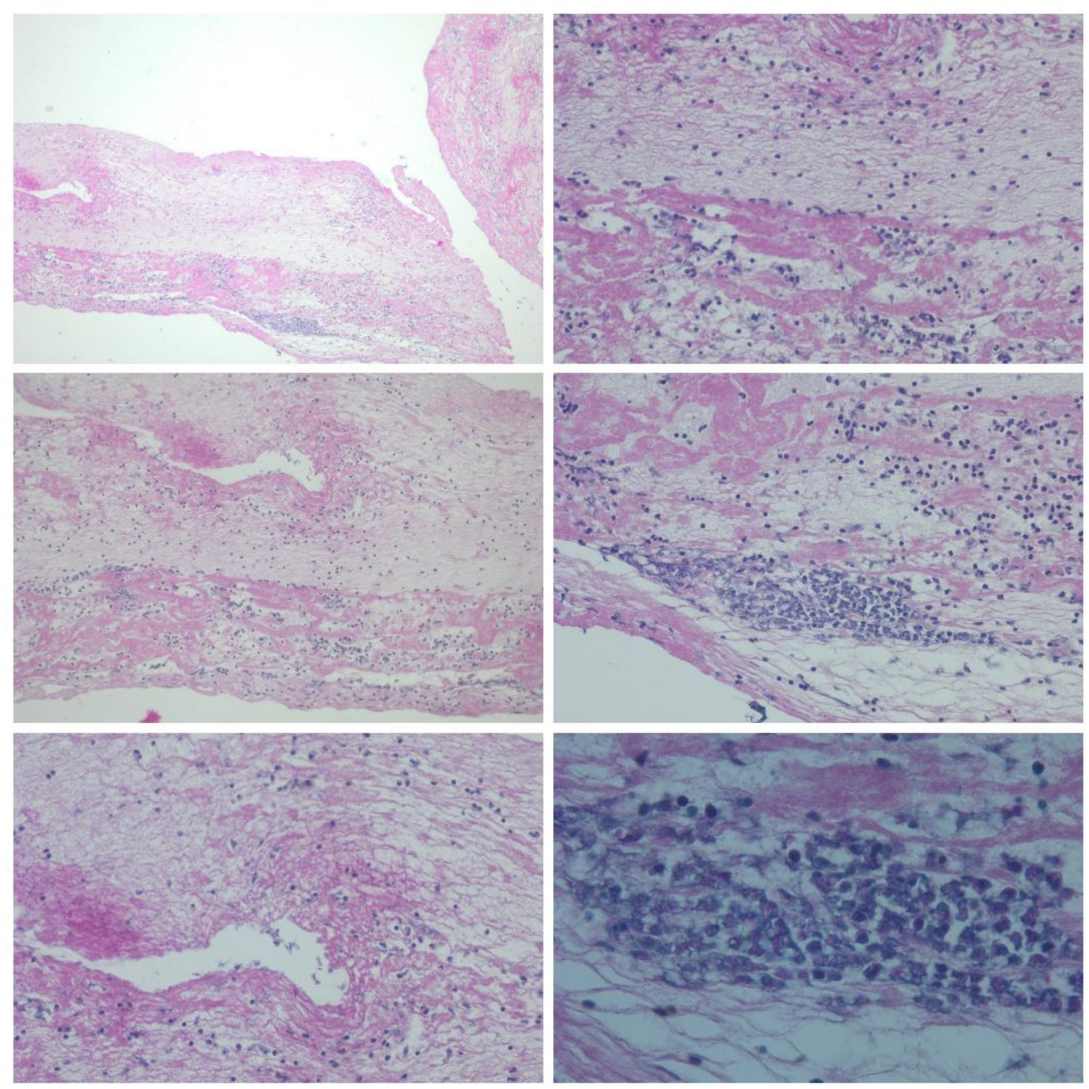
Histopathological images of a mixed-type thrombus (H&E staining) retrieved during mechanical thrombectomy. **(Upper row)** Cross-sections of the thrombus demonstrating a layered organization of thrombotic material, with numerous neutrophils localized in transitional zones between protein-rich and erythrocyte-rich regions. **(Middle row)** Thrombus fragments showing pronounced neutrophilic infiltration and focal nuclear degradation, consistent with early stages of neutrophil extracellular trap (NET) formation; dense accumulations of fibrous material with a mesh-like architecture are visible in the lower regions. **(Lower row)** High-magnification images documenting dense, irregular NET networks surrounding clusters of neutrophils and permeating the thrombus structure, indicating their role in stabilizing the thrombotic mass.

Thrombi from 30 patients were analyzed using the Histopathological methods (H&E and special stains; Figure 2). Results were consistent across methods and indicated that the thrombi were:

- Fibrin-rich (“white”), with a dense, multilayered network of fibrin and collagen
- Partially organized, containing numerous intrathrombus spaces
- Containing irregular, scattered neutrophil foci, indicative of their active role
- Poor in erythrocytes, distinguishing them from cardiogenic thrombi
- Devoid of atherosclerotic material, although exhibiting features of chronic vessel wall inflammation

These observations confirm the heterogeneity of the thrombus microenvironment and support further analyses of neutrophil phenotype and metabolic activity.

### 5.2 Hematological and clinical profile

Parallel arterial and peripheral blood samples were collected from 37 patients (Table 3). Observations included:

- Predominance of segmented granulocytes (77–79%)
- Lymphopenia
- High NLR (~7), stable between arterial and venous blood (ΔNLR ≈ −0.11)
- Altered DLR, MLR, and LMR indices typical of the acute phase of stroke

**Table 3:** Demographic, hematological, and clinical characteristics of patients undergoing endovascular treatment for acute ischemic stroke.

**Category/variable**	***N***	**Mean (SD) or *n* (%)**	**95% CI**
**Demographics**			
Age (years)	37	74.16 (11.65)	70, 78
Sex	37		
Female		25 (67.57%)	50%, 81%
Male		12 (32.43%)	19%, 50%
**Arterial blood leukogram parameters (%)**			
Segmented granulocytes	37	79.38 (10.05)	76, 83
Band granulocytes	37	1.59 (2.87)	0.64, 2.6
Lymphocytes	37	15.32 (8.67)	12, 18
Reactive lymphocytes	37	0.95 (1.56)	0.42, 1.5
Monocytes	37	1.57 (1.63)	1.0, 2.1
Basophils	37	0.38 (0.79)	0.11, 0.64
Eosinophils	37	0.30 (0.62)	0.09, 0.50
Other	37	0.30 (0.62)	0.09, 0.50
NLR	36	6.90 (3.92)	5.6, 8.2
DLR	16	54.75 (30.47)	39, 71
MLR	24	0.19 (0.15)	0.13, 0.26
LMR	25	9.06 (7.44)	6.0, 12
**Peripheral blood leukogram parameters (%)**			
Segmented granulocytes	37	76.65 (11.72)	73, 81
Band granulocytes	37	1.59 (2.44)	0.78, 2.4
Lymphocytes	37	17.76 (9.97)	14, 21
Reactive lymphocytes	37	1.11 (1.47)	0.62, 1.6
Monocytes	37	2.30 (2.21)	1.6, 3.0
Basophils	37	0.30 (0.62)	0.09, 0.50
Eosinophils	37	0.24 (0.49)	0.08, 0.41
Other	36	0.03 (0.17)	0.00, 0.08
NLR	37	6.99 (5.85)	5.0, 8.9
DLR	20	53.79 (28.98)	40, 67
MLR	28	0.23 (0.29)	0.12, 0.34
LMR	28	7.99 (4.81)	6.1, 9.9
ΔNLR	36	−0.11 (4.33)	−1.6, 1.4
**Stroke severity and imaging parameters**			
NIHSS at admission	11	14.45 (5.34)	11, 18
NIHSS after procedure	11	7.82 (5.29)	4.3, 11
NIHSS at discharge	11	6.18 (5.29)	2.6, 9.7
Core volume Brainomix (mL)	12	26.83 (19.38)	15, 39
Core volume Rapid (mL)	12	15.00 (18.43)	3.3, 27
Penumbra volume Brainomix (mL)	12	125.17 (78.85)	75, 175
ASPECT Brainomix	10	8.00 (1.56)	6.9, 9.1
**Treatment details**			
mTICI after procedure	12		
2b		1 (8.33%)	0.44%, 40%
2c		3 (25.00%)	6.7%, 57%
3		8 (66.67%)	35%, 89%
Neutrophil band form ratio	22	1.22 (1.52)	0.54, 1.9
Intravenous thrombolysis	12	6 (50.00%)	25%, 75%
Time of r-tPA administration from symptom onset (min)	5	110.80 (21.26)	84, 137
Time of r-tPA administration from last seen well (min)	5	146.80 (66.56)	64, 229

Continuous variables are presented as mean (standard deviation) with 95% confidence intervals for the mean. Categorical variables are presented as count (percentage) with 95% confidence intervals for proportions. *N* represents the number of observations available for each variable; variations in *N* reflect missing data for specific parameters. NLR, neutrophil-to-lymphocyte ratio; DLR, derived lymphocyte ratio; MLR, monocyte-to-lymphocyte ratio; LMR, lymphocyte-to-monocyte ratio; ΔNLR, difference in NLR between arterial and peripheral blood. NIHSS, National Institutes of Health Stroke Scale; ASPECT, Alberta Stroke Program Early CT Score; mTICI, modified Thrombolysis in Cerebral Infarction; r-tPA, recombinant tissue plasminogen activator. Confidence intervals are approximate and based on standard methods (e.g., *t* test for means and Wilson method for proportions).

Clinical and imaging parameters were as follows:

- Mean NIHSS at admission: 14
- Post-procedure NIHSS: 7.8
- Complete reperfusion (mTICI 3): 66.7% of patients
- Infarct core volume: 15–27 mL
- Penumbra volume: ~125 mL
- ASPECTS score: mean 8 points

### 5.3 Correlation analysis of hematological and clinical outcomes in patients undergoing endovascular treatment for acute ischemic stroke

**Figure 3:**
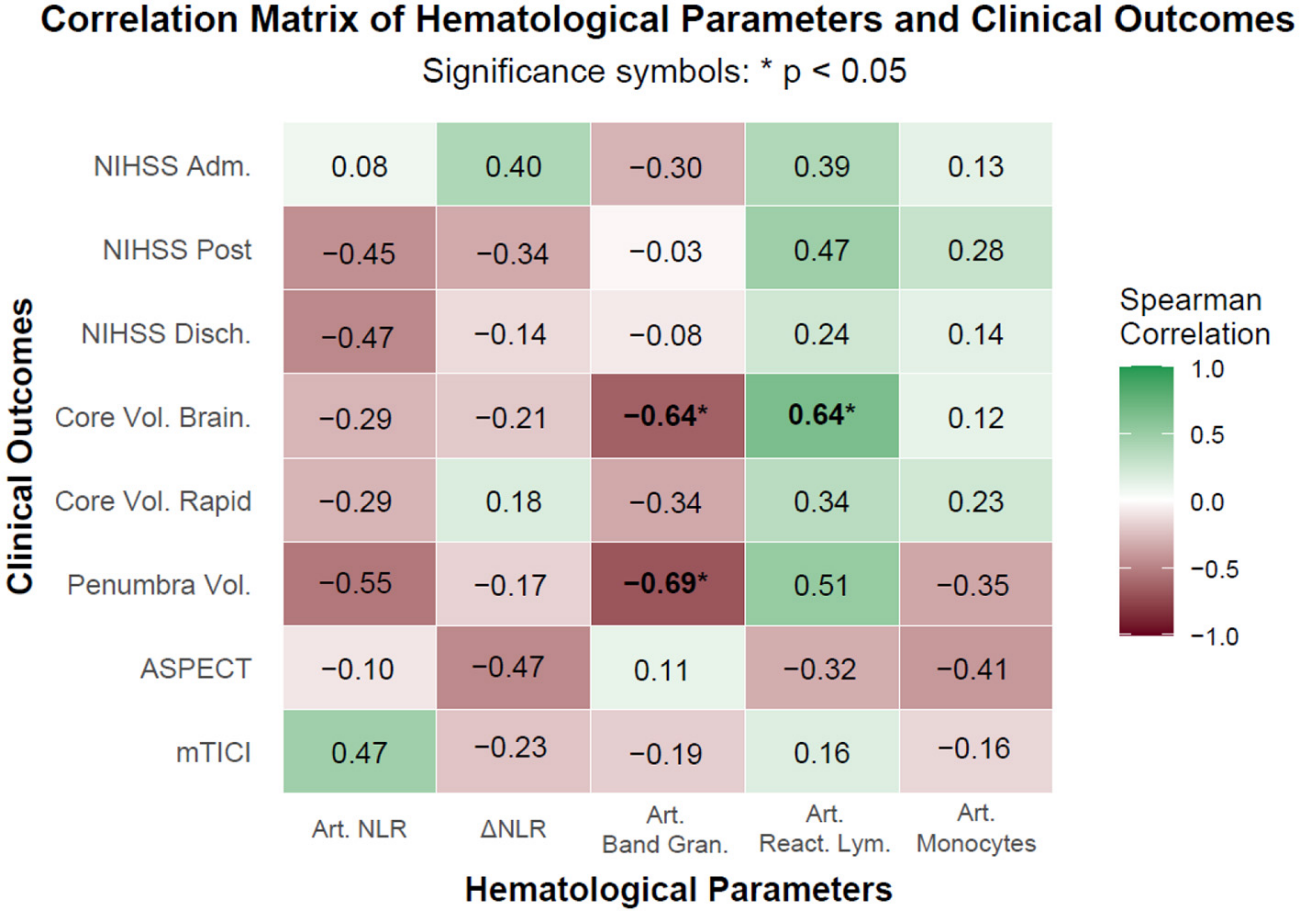
Spearman rank-order correlation map between arterial hematological parameters and clinical outcomes in patients undergoing endovascular treatment for acute ischemic stroke. Observed associations suggest that specific leukocyte subpopulations may influence infarct development and reperfusion outcomes. Notably, the negative correlation of band granulocytes with infarct core and penumbra volumes may indicate a potential protective effect, while the positive association of reactive lymphocytes with infarct volume may reflect increased tissue injury. The lack of statistical significance for several correlations, despite moderate effect sizes (e.g., reactive lymphocytes vs. post-procedure NIHSS), is likely due to the limited sample size. Larger cohort studies are warranted. Art NLR, Arterial Neutrophil-to-Lymphocyte Ratio (unitless); ΔNLR, Difference in NLR between Arterial and Peripheral Blood (unitless); Art. Band Gran., Arterial Band Granulocytes (%); Art. React. Lym., Arterial Reactive Lymphocytes (%); Art. Monocytes, Arterial Monocytes (%); NIHSS Adm, NIHSS at Admission (score); NIHSS Post, NIHSS after Procedure (score); NIHSS Disch., NIHSS at Discharge (score); Core Vol. Brain., Core Volume Brainomix (mL); Core Vol. Rapid, Core Volume Rapid (mL); Penumbra Vol., Penumbra Volume Brainomix (mL); ASPECT, ASPECT Brainomix (score); mTICI, mTICI after Procedure.

As illustrated in the correlation matrix (Figure 3), moderate negative correlations were observed between arterial band granulocytes and core infarct volume on Brainomix (ρ = −0.0.64, *p*= 0.024) as well as penumbra volume (ρ = −0.0.69, *p*= 0.013), both achieving statistical significance (*p* < 0.05). Conversely, arterial reactive lymphocytes showed a moderate positive correlation with Brainomix core volume (ρ = 0.64, *p*=0.026).

These findings reveal that higher proportions of band granulocytes may be associated with smaller infarct and penumbra volumes, potentially reflecting a protective or compensatory mechanism in the early phases of stroke, whereas elevated levels of reactive lymphocytes could indicate greater tissue damage. No other correlations reached statistical significance, including those involving arterial NLR or ΔNLR with NIHSS scores or mTICI, although several exhibited numerically moderate effect sizes (e.g., arterial NLR with NIHSS at discharge: ρ = −0.0.47, *p*= 0.140; arterial NLR with penumbra volume: ρ = −0.0.55, *p*= 0.071).

The estimated associations suggest that specific leukocyte subsets may influence infarct evolution and reperfusion outcomes, in line with previous evidence linking systemic inflammation to stroke pathophysiology. For example, the inverse relationship between band granulocytes and infarct volumes may inform future prognostic models, in which the release of immature neutrophils reflects varying degrees of ischemic burden. However, the absence of statistical significance in other pairings—despite moderate correlation coefficients (e.g., reactive lymphocytes with NIHSS post-procedure: ρ = 0.47, *p*= 0.140)-highlights the limited statistical power of the present analysis due to small sample sizes (e.g., *n*= 11 for NIHSS-related variables and *n*= 12 for imaging metrics). This constraint increases the risk of type II error and may obscure true biological associations, particularly for correlations exceeding |ρ| = 0.5. Consequently, larger, adequately powered studies will be essential to validate these preliminary observations and to clarify the mechanistic roles of these hematological parameters in post-stroke recovery.

### 5.4 Power analysis

A power analysis for detecting a correlation of ρ = 0.40, with α = 0.05 and 1–β = 0.80, indicated that at least 47 participants would be required. Allowing a 15% safety margin, a target sample size of 55 patients was adopted, consistent with preliminary data.

### 5.5 Key conclusions from preliminary analyses

- Thrombi exhibit a chronic, fibrin-rich phenotype with an active neutrophil population
- Hematological parameters are stable and suitable for translational analyses
- Specific leukocyte subpopulations may modulate infarct development
- Data support the hypothesis regarding the role of neutrophil immunometabolism in acute ischemic stroke (AIS)
- Power analysis confirms the adequacy of the planned cohort size

## 6 Conclusion

This statistical and methodological framework supports the implementation of the study: “*Comprehensive Immunological Profiling in Acute Ischemic Stroke: Myeloid Cell Activation and Molecular Signatures in Blood and Thrombus during Mechanical Thrombectomy.”* The combination of site-specific immune sampling, high-dimensional profiling, and rigorous data analysis is expected to yield novel insights into the immunopathology of acute ischemic stroke. The approach enables the identification of biomarkers predictive of stroke severity, therapeutic response, and long-term recovery, potentially guiding precision medicine strategies in stroke care.

## 7 Safety evaluation

Within this study, the only procedure exceeding routine clinical management is the collection of peripheral venous blood samples. This procedure involves minimal risk of minor adverse events (AEs), such as local pain, bruising, hematoma formation, minor bleeding, or local inflammatory reactions. These events are typically mild and self-limiting, requiring no additional interventions beyond standard care. All potential adverse events related to peripheral venous blood sampling will be systematically documented using a dedicated AE reporting form and classified according to severity (mild, moderate, severe), duration, and causality. Each AE will be categorized as unrelated, probably related, or related to the patient's participation in the research. Patients will be monitored during hospitalization and follow-up visits as part of standard clinical practice to identify potential delayed AEs (e.g., infection at the venipuncture site, delayed hematoma formation). Such events will also be included in the study documentation. In the unlikely event of a serious adverse event (SAE)—defined as any medical event resulting in death, hospitalization, permanent disability, or requiring significant medical intervention—the research team will immediately notify the Ethics Committee and consider withdrawing the patient from the study, prioritizing patient safety and welfare. Due to the observational nature and low-risk profile of this study, we deem the establishment of an independent Safety Review Board unnecessary. However, the principal investigators, supported by clinical neurologists and interventional radiologists, will critically review any ambiguous or severe AE cases to determine causality and manage patient safety effectively. The collection of arterial blood and thrombus material occurs routinely during standard mechanical thrombectomy procedures, and thus does not involve additional invasive interventions specific to this research. Consequently, any adverse events arising from these procedures would be attributable to standard therapeutic practice and not directly to research-specific activities.

## 8 Data collection and management

All relevant clinical and research data—including clinical parameters, imaging studies, procedural outcomes (such as NIHSS, mRS, and TICI scores), and laboratory/immunological results—will be initially collected using standardized paper-based Case Report Forms (CRFs), which serve as the primary data source, and subsequently entered into a dedicated, secure electronic case report form (eCRF) system developed specifically for this study.

Clinical data routinely generated during patient care (e.g., imaging findings, procedural results, and clinical assessments) will be extracted from the hospital's electronic medical information system by trained and authorized study personnel. These data will be pseudonymized using unique subject identification codes before being recorded in the paper CRFs and later transferred into the eCRF.

Research-specific data generated through external laboratory analyses (e.g., immunological profiling, transcriptomics, microscopy) will be directly recorded in the eCRF by authorized personnel. All entries in the electronic system will be subject to audit trails to ensure traceability and data integrity.

The electronic database will be stored on encrypted, password-protected computers located on secure institutional servers, with access strictly limited to designated members of the research team, including data managers and statisticians. Regular data backups will be performed to prevent data loss, and access logs will be maintained to monitor any changes.

All physical (paper-based) documentation containing sensitive or identifiable patient information (e.g., signed consent forms, clinical reports) will be securely stored in a locked cabinet located in the office of Prof. Tadeusz Popiela. Access to this cabinet will be restricted to authorized personnel only.

Both physical and electronic records will be securely archived for a minimum of 5 years following the final publication of the study results, in accordance with institutional policies and applicable legal regulations, including the General Data Protection Regulation (GDPR).

Regular internal data audits will be conducted throughout the study to ensure data accuracy, completeness, and compliance with ethical, regulatory, and data protection standards.

## 9 Quality control

To ensure consistency and high quality of collected data and analytical results, all study procedures—including blood and thrombus collection, sample processing, immunological assays, and molecular analyses—will be performed according to standardized operating procedures (SOPs) developed prior to study initiation. All personnel involved in sample acquisition and laboratory analyses will receive detailed practical training focused specifically on methodological aspects relevant to the study, such as sample stabilization, handling, labeling, transport, and advanced analytical techniques (spectral flow cytometry, RNA sequencing, microscopy techniques). Completion of training will be documented, and adherence to SOPs will be periodically verified. Prior to initiating the main phase of the study, pilot validation tests will be conducted using reference material to assess the accuracy, reproducibility, and analytical consistency of key laboratory procedures. To assess measurement consistency and reliability, the study protocol will include both intra-operator and inter-operator variability tests. Intra-operator reproducibility will be evaluated through repeated measurements performed by the same investigator on selected samples. Inter-operator reproducibility will be assessed by comparing results obtained independently by different investigators. Intraclass correlation coefficients (ICC) will be calculated separately for cytometric parameters (e.g., CD11b, CD66b expression), cytokine levels, RNA expression profiles, and cfDNA quantification. Throughout the duration of the study, the principal investigator will conduct regular review meetings with the research team to monitor study progress, address potential methodological issues, and verify data integrity. Additionally, periodic audits will be carried out by an independent Data Monitoring Committee to ensure compliance with ethical standards, study protocols, and good clinical practice (GCP) guidelines. All costs related to study-specific procedures, including laboratory analyses, sample processing, and additional tests required for the purposes of this research, will be covered by the research grant funding the project.

## 10 Outcome assessment and bias control

Angiographic reperfusion (TICI) is independently assessed by two interventional neuroradiologists blinded to immunological data. Functional outcomes at 90 days (mRS) are evaluated by certified neurologists not involved in the thrombectomy procedure, minimizing assessment bias.

## 11 Study limitations and project risks

This study has several inherent limitations and risks that should be considered when interpreting the results.

First, the single-center design and the intrinsic heterogeneity of thrombus composition limit the generalizability of the findings. Sampling variability, partial clot fragmentation during retrieval, and the limited availability of arterial blood in some cases may introduce bias. Moreover, arterial blood sampling at the site of occlusion was feasible only in patients with successful thrombus retrieval, potentially introducing selection bias toward individuals with more favorable anatomical or procedural characteristics. While the exclusion of IVT-treated patients may reduce generalizability, it enhances the interpretability of local immune signatures.

Technical risks include RNA degradation, hemolysis, and limited thrombus quantity. These risks are mitigated through strict collection protocols, immediate sample processing, and rigorous quality control.

Isolation-related risks, such as cell damage or ex vivo activation, are addressed by employing gentle dissociation procedures, completing cell isolation within 60 minutes, and validating markers of cell integrity and phenotype.

Biological variability, including etiological heterogeneity and confounding effects of medications (e.g., antiplatelet agents), represents an additional challenge. Stratification according to TOAST classification and the use of multivariable models help to minimize these confounding influences.

Animal model studies face risks such as variability in MCAO models and mortality in transgenic lines. These are mitigated through strict standardization of procedures and careful monitoring of cerebral blood flow.

Analytical risks include the integration of multidimensional data and the potential overinterpretation of observed correlations. These are addressed through mechanistic validation in animal models and rigorous statistical control.

Taken together, these limitations and risks highlight the complexity of translational thrombus research and underscore the need for careful interpretation of findings, replication in larger and multi-center cohorts, and complementary mechanistic studies.

## 12 Summary

This study represents a novel, translational investigation into the immune mechanisms underlying acute ischemic stroke. By combining direct sampling from the site of vascular occlusion with detailed immunological profiling and clinical correlation, the project aims to identify actionable immune biomarkers and pathways—particularly the MIF–RIPK1 axis—that influence stroke severity and response to treatment. The integrative design ensures scientific rigor, clinical relevance, and potential translational impact in the development of personalized stroke therapies.
